# Comprehensive Analysis of a Nine-Gene Signature Related to Tumor Microenvironment in Lung Adenocarcinoma

**DOI:** 10.3389/fcell.2021.700607

**Published:** 2021-09-01

**Authors:** Haihui Zhong, Jie Wang, Yaru Zhu, Yefeng Shen

**Affiliations:** ^1^Department of Thoracic Surgery, Meizhou People’s Hospital (Huangtang Hospital), Meizhou Hospital Affiliated to Sun Yat-sen University, Meizhou Academy of Medical Sciences, Meizhou, China; ^2^Institute for Pathology, University Hospital of Cologne, Cologne, Germany; ^3^Department of Critical Care Medicine, Zhujiang Hospital, Southern Medical University, Guangzhou, China

**Keywords:** tumor microenvironment, lung adenocarcinoma, biomarker, diagnosis, prognosis

## Abstract

Lung adenocarcinoma (LUAD) is the most common malignancy, leading to more than 1 million related deaths each year. Due to low long-term survival rates, the exploration of molecular mechanisms underlying LUAD progression and novel prognostic predictors is urgently needed to improve LUAD treatment. In our study, cancer-specific differentially expressed genes (DEGs) were identified using the robust rank aggregation (RRA) method between tumor and normal tissues from six Gene Expression Omnibus databases (GSE43458, GSE62949, GSE68465, GSE115002, GSE116959, and GSE118370), followed by a selection of prognostic modules using weighted gene co-expression network analysis. Univariate Cox regression, least absolute shrinkage and selection operator (LASSO), and multivariate Cox regression analyses were applied to identify nine hub genes (*CBFA2T3, CR2, SEL1L3, TM6SF1, TSPAN32, ITGA6, MAPK11, RASA3, and TLR6*) that constructed a prognostic risk model. The RNA expressions of nine hub genes were validated in tumor and normal tissues by RNA-sequencing and single-cell RNA-sequencing, while immunohistochemistry staining from the Human Protein Atlas database showed consistent results in the protein levels. The risk model revealed that high-risk patients were associated with poor prognoses, including advanced stages and low survival rates. Furthermore, a multivariate Cox regression analysis suggested that the prognostic risk model could be an independent prognostic factor for LUAD patients. A nomogram that incorporated the signature and clinical features was additionally built for prognostic prediction. Moreover, the levels of hub genes were related to immune cell infiltration in LUAD microenvironments. A CMap analysis identified 13 small molecule drugs as potential agents based on the risk model for LUAD treatment. Thus, we identified a prognostic risk model including CBFA2T3, CR2, SEL1L3, TM6SF1, TSPAN32, ITGA6, MAPK11, RASA3, and TLR6 as novel biomarkers and validated their prognostic and predicted values for LUAD.

## Introduction

Lung cancer—with 1,800,000 new lung cancer cases worldwide each year—is the most malignant cancer in males and females (Torre et al., 2015; Sung et al., 2021). Lung adenocarcinoma (LUAD), the most common histological subtype of non-small-cell lung cancer, accounts for approximately 40% of lung cancer patients. Although significant strides have been made in recent decades, including surgical resection, chemotherapy, radiation therapy, and immune-based therapies, the long-term survival rate of LUAD patients remains unsatisfactory (Walder and O’Brien, 2017). One possible reason is that only less than 25% of LUAD patients harbor druggable molecular mutations, including EGFR, BRAF V600E, MET, and ALK, resulting in there being no possibility of the receipt of the targeted therapy for the majority of LUADs (Cancer Genome Atlas Research Network, 2014; Singal et al., 2019; Cavagna et al., 2021; Nassar et al., 2021). Currently, an improved understanding of the molecular mechanisms involved in tumorigenesis and the exploration of biomarkers are essential to improve the survival rates of LUAD patients.

Recently, many biomarkers have been reported as playing a critical role in oncogenicity and providing potential options for targeted therapies (Xie et al., 2019; Hwang et al., 2020). Complement C7, a newly detected tumor suppressor, has been revealed to be highly associated with clinical features and immune infiltration levels, presenting a strong therapeutic potential for prostate cancer treatment (Chen et al., 2020a). Furthermore, acetyl-CoA acetyltransferase inhibits the proliferation and migration of clear cell renal cell carcinoma cells *in vitro* and has been validated as a prognostic and progression biomarker via significant correlation with overall survival (OS) and disease-free survival rates (Chen et al., 2019). In addition, it was uncovered a nine-gene signature comprising MET, KLK10, COL17A1, CEP55, ANKRD22, ITGB6, ARNTL2, MCOLN3, and SLC25A45 has been identified as predicting the survival of pancreatic cancer (Wu M. et al., 2019), providing possible therapies. Hence, it is urgent to discover molecular markers highly associated with survival to contribute to improving the effect of targeted therapeutic approaches.

In this study analyzing the gene expression profiles of six Gene Expression Omnibus (GEO) datasets, we identified 1,219 differentially expressed genes (DEGs) between LUAD tumors and normal tissues. A weighted gene co-expression network (WGCNA) network analysis was conducted to explore the module related to clinical traits. Univariate Cox regression, least absolute shrinkage and selection operator (LASSO), and multivariate cox regression analyses revealed nine key genes highly associated with the LUAD prognosis. Moreover, a prognostic risk model was built on hub genes levels, acting as an independent factor for LUAD prognosis. It was indicated that the risk model and nine hub genes were correlated with immune cell infiltration. Additionally, potential small molecular drugs were detected for the possible targeted therapy. Thus, our findings suggested a prognostic risk model including CBFA2T3, CR2, SEL1L3, TM6SF1, TSPAN32, ITGA6, MAPK11, RASA3, and TLR6 serves as a novel biomarker and uncovered their prognostic and predictive values to provide molecular evidence of new potential clinical strategies for LUAD.

## Materials and Methods

### Collection of Data

The LUAD RNA expression profile and corresponding clinical characteristics were obtained from The Cancer Genome Atlas (TCGA) (Tomczak et al., 2015). Specifically, our work contained 585 LUAD samples and 594 RNA-sequencing data. Datasets met the following criteria were eligible: (1) the microarray data should include mRNA; (2) datasets included the data from human LUAD and adjacent normal tissues; (3) a minimum of 5 pairs of tissues was enrolled. Meanwhile, six eligible microarray datasets [GSE43458 (Kabbout et al., 2013), GSE62949 (Mansfield et al., 2015), GSE68465 (Director’s Challenge Consortium for the Molecular Classification of Lung et al., 2008), GSE115002 (Cui et al., 2019), GSE116959 (Moreno Leon et al., 2019), and GSE118370 (Xu et al., 2018)] were obtained from GEO databases for the expression of mRNA in LUAD patients. Single-cell transcriptome file of GSE149655 was obtained from GEO database (Dost et al., 2020).

### Identification of Robust Differentially Expressed Genes

The R package “limma” was utilized to normalize the data and analyze DEGs based on the series matrix files of datasets (Ritchie et al., 2015). Six GSE datasets were then combined and filtrated by a robust rank aggregation (RRA) (Kolde et al., 2012), and DEGs met the criteria of |log 2-fold change (FC)| > 0.8 and FDR (False Discovery Rate) -adjust P value <0.05. The R package “OmicCircos” was applied to visualize the locations of DEGs in the chromosome. Moreover, “Seurat” package in R was used for quality control, statistical analysis, and exploration of the single- cell RNA-seq data. The batch effect from studies was removed with regularized negative binomial regression by the “Seurat” package (Butler et al., 2018). Non-linear dimensional reduction was performed with the UMAP method. Cluster biomarkers were found by the “Seurat” package.

### Gene Ontology and Kyoto Encyclopedia of Genes and Genomes Pathway Analyses

We performed gene ontology (GO) enrichment analysis comprised of biological process (BP), cellular components (CC), molecular function (MF), and Kyoto Encyclopedia of Genes and Genomes (KEGG) pathway analysis for DEGs to explore the high-level functions and utilities of the biological system by using the R package “clusterProfiler” (Harris et al., 2004; Kanehisa et al., 2016).

### Weighted Gene Co-expression Network and Key Module Identification

We selected the top low p-value 4000 genes based on the results of RRA to construct a co-expression network with the weighted gene co-expression network (“WGCNA”) package in R, determining the clinical trait–related modules and hub genes among the DEGs. The samples were clustered by a hierarchical clustering after outliers were eliminated at the threshold of 90 and minimal number of samples were 50. A soft-threshold power with a scale-free R^2^ above 0.9. The unsigned network was built with blockwiseModules function with “WGCNA” package, which was set the soft-threshold power as 4, cut height as 0.25, and the minimal module size as 30 for network construction and module detection. The module with the highest correlation with LUAD was considered the key module. Hub genes were analyzed with softConnectivity function by “WGCNA” package in R software.

### Construction and Validation of Hub Genes and the Prognostic Model

A univariate regression analysis was performed to identify the potential prognostic genes. To detect key genes for the construction of a prognostic model, glmnet from the R software package was used for LASSO, and multivariate regression analysis were employed. The coefficient was analyzed by the survival coxph and step function of “survival” package in R. The risk scores for LUAD patients were calculated with the mRNA levels weighted by the estimated regression coefficient in the multiple Cox regression analysis. Meanwhile, univariate and multivariate regression analyses were applied to determine the independent prognostic factors for LUAD patients. A receiver operating characteristic (ROC) analysis was used to estimate the accuracy and clinical utility of the model for prognosis.

### Validation of Protein Expressions of Hub Genes

To detect the protein expression of the hub genes, the Human Protein Atlas (HPA) database provided the immunohistochemistry results for LUAD tumors and normal tissues.

### Mutation Profiles

The cBio cancer genomics portal (cBioPortal^1^) is a tool that analyzes genomic alterations from various cancer samples (Cerami et al., 2012). We investigated the mutation landscape of genes in LUAD.

### Construction and Evaluation of a Predictive Nomogram

Nomogram and calibrate curves were established with the “rms” package in R software to identify independent predictive factors. The validation of the sensitivity and specificity of the nomogram in predicting OS was detected by the ROC curve analysis.

### Correlation Between Gene Expression and Immune Cell Infiltration

The tool Cell-type Identification By Estimating Relative Subsets Of RNA Transcripts (CIBERSORT) was applied to investigate the correlation between these hub genes and 22 immune cells. We examined the correlation between expression of the hub genes and tumor-infiltrating immune cells by TIMER^2^, which included different types of cancer samples accessible in the TCGA cohort.

### Identification of Small Molecular Drugs

Connectivity Map (CMap) was applied to access molecule drugs highly associated with certain diseases (Lamb et al., 2006). The small molecule drugs, meeting the criteria of |mean| ≥ 0.40, instances (*n*) ≥ 5, and *P* < 0.05, were considered as potential treatments for LUAD patients.

### Statistical Analysis

All the values were presented as means ± the standard deviation (SD). The *t*-test together with a one-way analysis of variance was applied to assess the differences in the two groups. A value of *P* < 0.05 was considered a significant difference. All calculations were performed using R software.

## Results

### Identification and Chromosome Locations of DEGs

**FIGURE 1:**
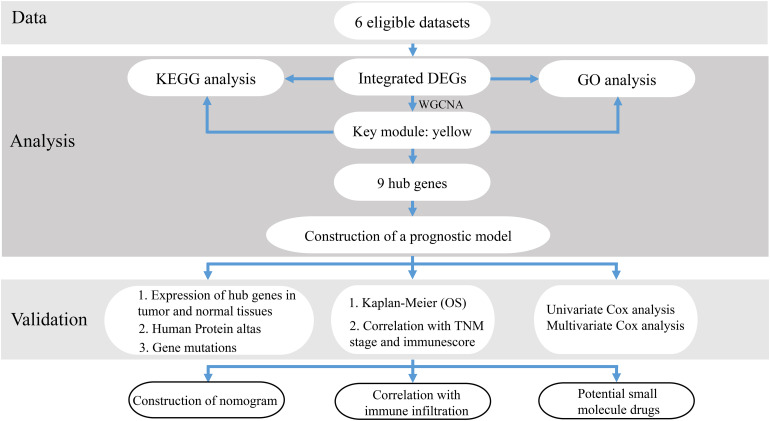
The workflow of this study.

**FIGURE 2:**
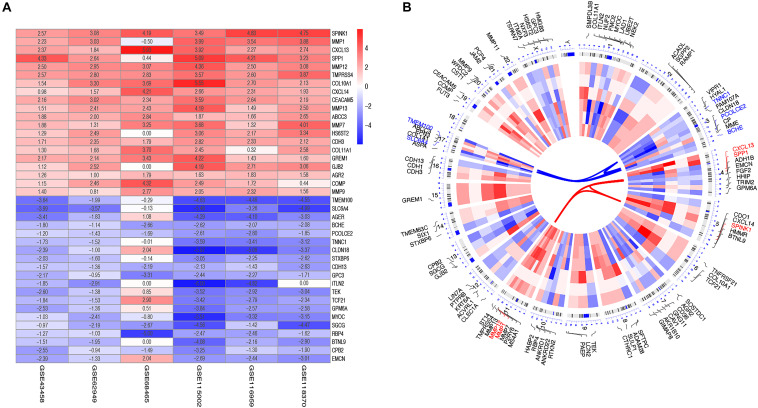
Identification and chromosomal positions of DEGs in the integrated microarray analysis. **(A)** Heatmap showed the top 20 upregulated and downregulated DEGs in GEO series accessions. Each row denoted one DEG and each column represented one dataset. The color changed from red to blue indicated the dysregulation from up to down. The numbers in the box standed for logarithmic fold change; **(B)** Circos plot of expression patterns and chromosomal positions of top DEGs. The outer circle represented chromosomes, and lines coming from each gene pointed to their specific chromosomal locations. GSE43458, GSE62949, GSE68465, GSE115002, GSE116959, and GSE118370 were presented from the outside to the inside. The red lines in the inner layer indicated adjusted P-value of each gene. According to adjusted P, the top five up-regulated genes (red) and the top five down-regulated ones (blue) were connected with red and blue lines in the center of the Circos plot.

After reviewing the GEO database, six eligible microarray datasets (GSE43458, GSE62949, GSE68465, GSE115002, GSE116959, and GSE118370) were included in our study, and the workflow is shown in Figure 1. The main characteristics of included GEO datasets are summarized in Table 1. There were 665 LUAD and 141 normal tissues analyzed in our work to explore the DEGs. Based on the results of the RRA analysis with |log 2-fold change (FC)| > 0.8 and adjust P value < 0.05, 1,219 significant DEGs, including 496 upregulated and 723 downregulated, were identified (Supplementary File 1 and Supplementary Figure 1). SPINK1 ranked first among all upregulated genes (*P* = 2E-17, adjusted *P* = 8.69E-13), while TMEM100 (*P* = 1.42E-14, adjusted *P* = 3.45E-10) was the most significant downregulated gene in LUAD tissues. Moreover, the top 20 upregulated and downregulated DEGs from the six datasets were shown on a heatmap (Figure 2A).

**TABLE 1:** Characteristics of the included datasets.

Series accession ID	Country	Number of samples		Analyzed genes	Platform ID
		Tumor	Normal		
GSE43458	United States	80	30	23305	GPL6244
GSE62949	United States	28	28	15562	GPL8432
GSE68465	United States	442	14	12548	GPL96
GSE115002	China	52	52	21752	GPL13497
GSE116959	France	57	11	32077	GPL17077
GSE118370	China	6	6	21653	GPL570

The top 50 upregulated and downregulated genes were selected to visualize their expression patterns and chromosomal locations (Figure 2B). The top five upregulated genes (SPINk1, MMP1, CXCL13, SPP1, and MMP12) were located in chromosomes 5, 11, 4, 4, and 11. Meanwhile, the top five downregulated genes (TMEM100, SLC6A4, BCHE, PCOLCE2, and TNNC1) were distributed in chromosomes 17, 17, 3, 3, and 3.

### Enrichment Analysis of DEGs

**FIGURE 3:**
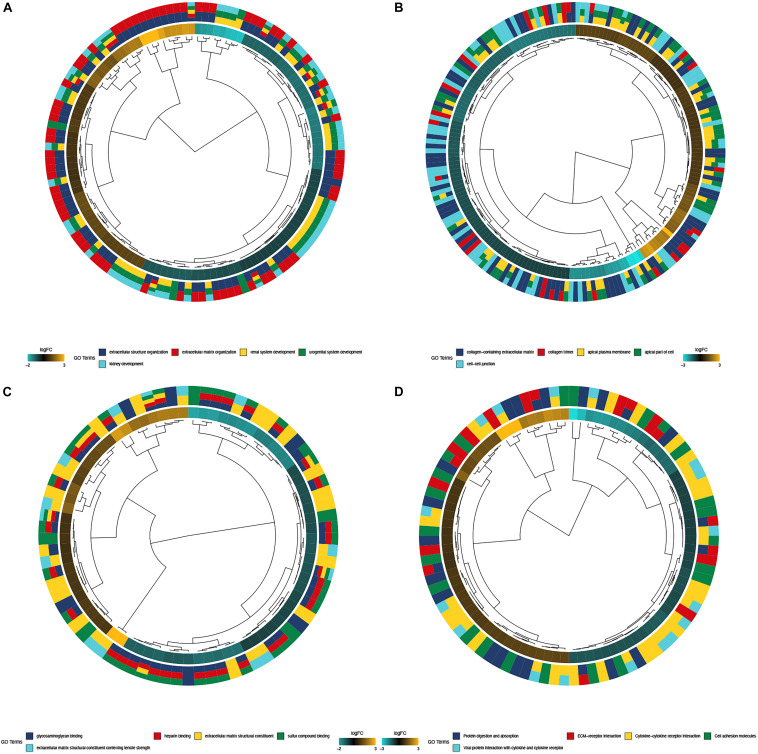
Functional enrichment analysis of top 300 DEGs. **(A)** BP of GO analysis; The outer circle was a bar plot where the height of the bar indicated the significance of GO terms. The inner ring showed a scatter plot of the expression of DEGs in each enriched gene ontology term; **(B)** CC of GO analysis, **(C)** MF of GO analysis; **(D)** Top 5 enriched KEGG pathways for DEGs.

The biological functions of DEGs were explored using GO annotation. Enriched BPs were extracellular structure organization, extracellular matrix organization, renal system development, urogenital system development, and kidney development (Figure 3A). Concerning CC, there were collagen-containing extracellular matrix, collagen trimer, apical plasma membrane, the apical part of the cell, and the cell-cell junction (Figure 3B). In terms of MF, the DEGs were enriched in glycosaminoglycan binding, heparin binding, extracellular matrix structural constituent, sulfur compound binding, and extracellular matrix structural constituent conferring tensile strength (Figure 3C). Additionally, enriched KEGG pathways were protein digestion and absorption, ECM (extracellular matrix) -receptor interaction, cytokine-cytokine receptor interaction, cell adhesion molecules, and viral protein interaction with the cytokine and cytokine receptor (Figure 3D).

### WGCNA and Key Module Identification

**FIGURE 4:**
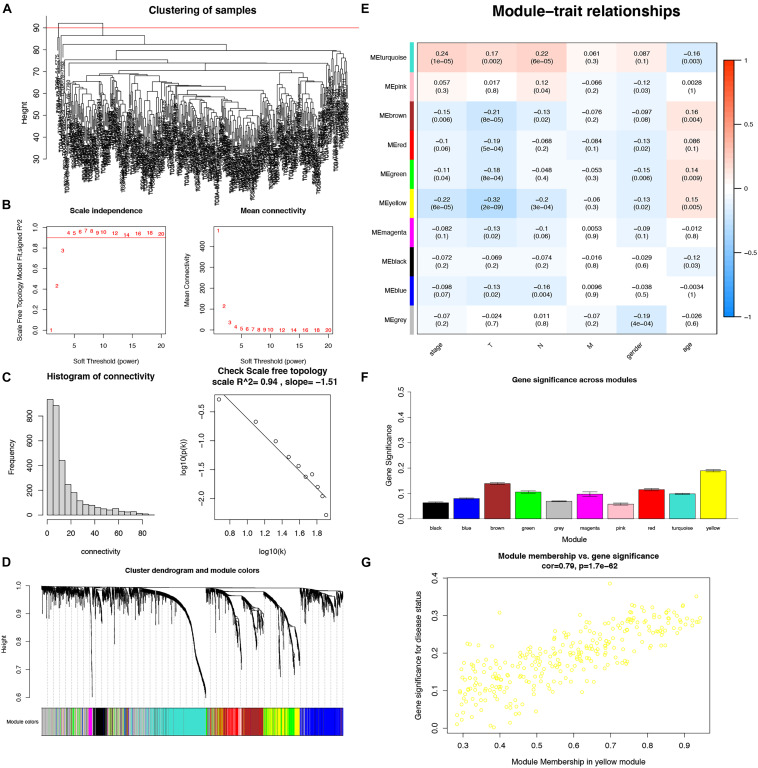
Identification of key modules associated with clinical traits in the TCGA-LUAD dataset by WGCNA. **(A)** Clustering dendrograms of samples; **(B)** Analysis of the scale-free fit index and the mean connectivity for various soft-thresholding powers; **(C)** Checking the scale-free topology when β = 4; **(D)** Dendrogram of all DEGs clustered with dissimilarity measure based on topological overlap; **(E)** Heatmap of the correlation between module eigengenes and clinical traits. Each row denoted a module eigengene, each column represented a clinical trait and each cell contained the correlation coefficient and P-value; **(F)** Gene significance in different modules (bottom); **(G)** Scatter plot of genes in yellow module.

To detect the key modules highly associated with clinical traits of LUADs, a WGCNA was conducted on the TCGA-LUAD dataset incorporating the DEGs (Figure 4A). By setting the soft-threshold power as 4 (scale-free *R*^2^ = 0.94, slope = −1.51; Figures 4B,C). A total of 30 modules were acquired from the co-expression network after merging similar modules according to a cut height of 0.25 (Figure 4D). According to a heatmap of module–trait correlations, we considered that the yellow module containing 287 DEGs was the most negatively correlated with clinical traits, particularly including the stage (correlation coefficient = −0.22, *P* = 6E−05) and T (correlation coefficient = −0.32, *P* = 2E−09) (Figure 4E and Supplementary File 2). Additionally, the module significance of the yellow module was higher compared with other ones, implying there was a significant correlation with T (Figure 4F). Moreover, the correlation and p-value between the module membership and gene significance values were 0.79 and 1.7E-62, respectively (Figure 4G). Thus, the yellow module was the most negative module with clinical traits.

### Functional Enrichment Analysis of the Yellow Module

**FIGURE 5:**
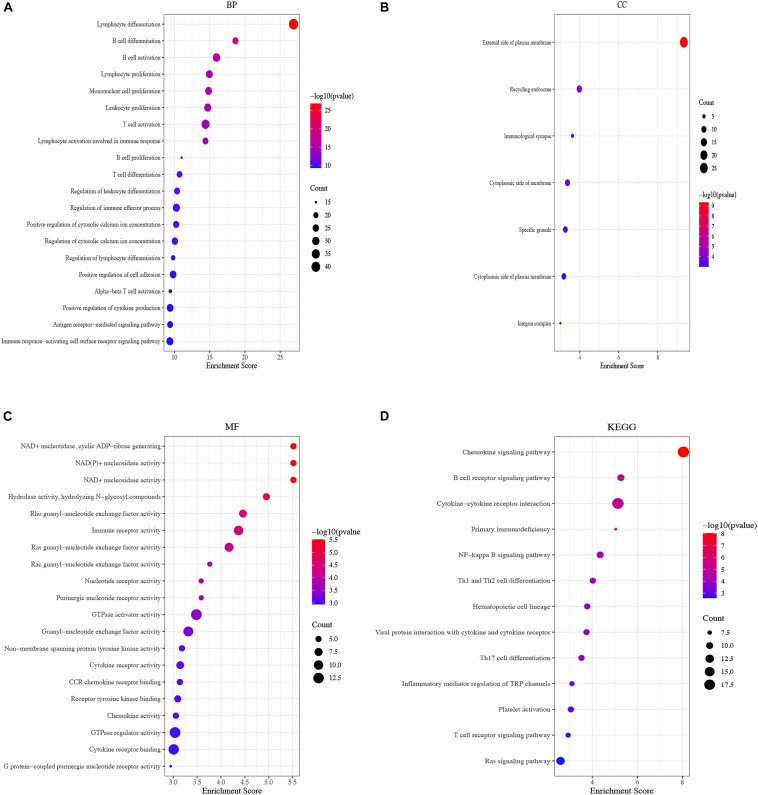
Functional enrichment of the yellow module. **(A)** Bubble plots of BP of GO analysis; **(B)** Bubble plots of CC of GO analysis, **(C)** Bubble plots of MF of GO analysis, **(D)** Bubble plots of KEGG pathways.

To further explore the biological functions of DEGs from the yellow module, GO annotation was conducted. The top BP enrichment terms were “lymphocyte differentiation,” “B cell differentiation” and “B cell activation” (Figure 5A). Concerning CC were “external side of plasma membrane,” “recycling endosome” and “immunological synapse” (Figure 5B). In terms of MF, the DEGs were “enriched in NAD + nucleotidase,” “cyclic ADP-ribose generating,” “NAD(P) + nucleosidase activity” and “NAD + nucleosidase” (Figure 5C). Additionally, KEGG pathways showed that DEGs were highly enriched in the chemokine signaling pathway, followed by the B cell receptor signaling pathway and cytokine-cytokine receptor interaction (Figure 5D).

### Establishment of a Prognostic Risk Model

**FIGURE 6:**
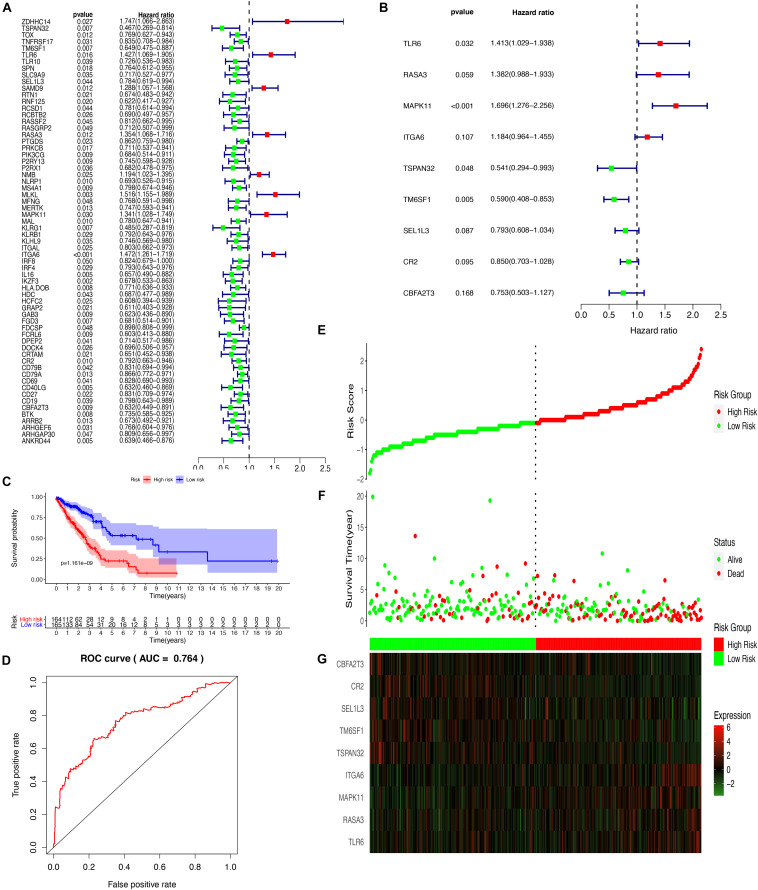
Establishment and assessment of the prognostic risk model. **(A)** Forest plot of 9 hub genes related to the survival of LUADs analyzed by univariate cox regression; **(B)** Forest plot of 9 hub genes analyzed by multivariate cox regression **(C)** The Kaplan-Meier curve of the prognostic model; **(D)** The ROC curve for assessing the reliability of the prognostic model; Distribution of risk score **(E)**, survival status **(F)**, and the 9 genes expression **(G)**.

To more ideally reveal the prognostic value of DEGs from the yellow module in LUAD, the correlation of the mRNA levels and the prognosis was explored after a univariate Cox regression analysis with the cut-off criteria of *P* < 0.05, resulting in 64 DEGs with *P* < 0.05 from the yellow module (Figure 6A). The prognosis-related genes were further analyzed with a LASSO Cox regression algorithm from the expression of TCGA-LUAD and normal tissues (Supplementary Figure 2). To further select the DEGs with the greatest prognostic value, a multiple stepwise Cox regression was conducted to determine their importance, and nine key genes (CBFA2T3, CR2, SEL1L3, TM6SF1, TSPAN32, ITGA6, MAPK11, RASA3, and TLR6) were obtained consisting of the prognostic signature (Figure 6B). The LUAD patients from TCGA were divided into high- and low-risk groups based on the median level of the risk score. The formula of calculating risk scores was as follows: -0.283625763 ^∗^ CBFA2T3 – 0.162111327 ^∗^ CR2 – 0.231743871 ^∗^ SEL1L3 -0.527168072 ^∗^ TM6SF1– 0.614785144 ^∗^ TSPAN32 – 0.169271203 ^∗^ ITGA6 + 0.528539595 ^∗^ MAPK11 + 0.323227079 ^∗^ RASA3 + 0.3453755 ^∗^ TLR6. A Kaplan–Meier survival analysis from this model detected that the patients in the high-risk group resulted in poor prognostic outcomes compared with those in the low-risk group (Figure 6C). To determine the predictive accuracy of the 5-year OS ROC curves were built, and the area under the curve (AUC) value was 0.764 (Figure 6D). Risk score, survival status, and each gene in the formula in LUAD patients were additionally analyzed (Figures 6E–G). To validate the reliability of the risk model from the TCGA-LUAD, we determined the model with a GSE68465 dataset, which suggested that patients with high-risk scores suffered from higher mortality rates than low-risk score patients (Supplementary Figure 3).

### Validation of Hub Genes

**FIGURE 7:**
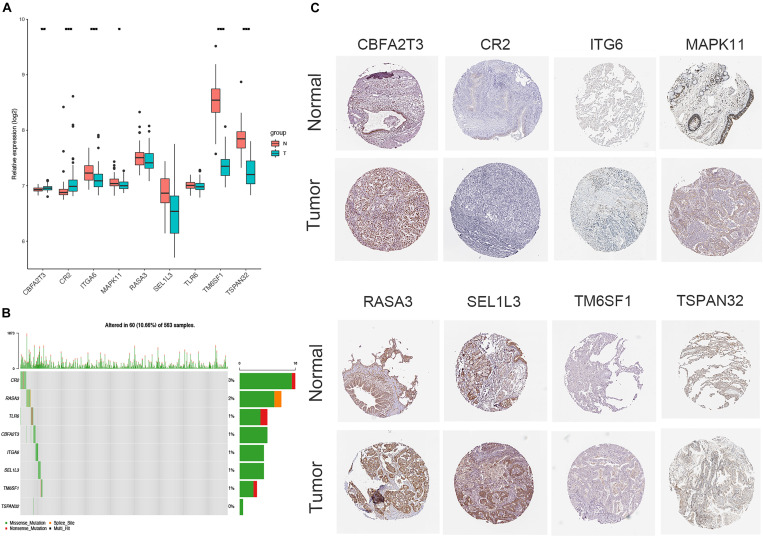
Verification of the expression and genetic alterations of 9 hub genes in tumor and normal tissues. **(A)** The boxplot showed the expression of 9 hub genes between tumor and normal tissues in TCGA database; **(B)** The protein levels of hub genes were presented by immunohistochemical staining analysis from Human Protein Atlas database; **(C)** A visual summary across a set of LUAD from TCGA showed the genetic alterations connected with the 9 hub genes which were altered in 60 (10.66%) of 536 sequenced patients.

The expression of CBFA2T3, CR2, ITGA6, MAPK11, TM6SF1, and TSPAN32 were significantly higher LUAD samples compared to normal tissues, while no difference existed in the levels of RASA3, SEL1L3, and TLR6 between LUAD and normal tissues (Figure 7A). The correlation of the expression of hub genes and the tumor-node-metastasis (TNM) stage is shown in Supplementary Figure 4. Additionally, immunohistochemistry staining obtained from HPA determined the consistent protein levels of eight other hub genes without TLR6 due to the lack of staining on HPA (Figure 7B). In order to analyze genomic alternations, we measured the alteration rates based on the cBioPortal. There were 10.66% (60/563) genetic alterations totally, and CR2 was the most common alteration (3%) in LUAD patients (Figure 7C).

**FIGURE 8:**
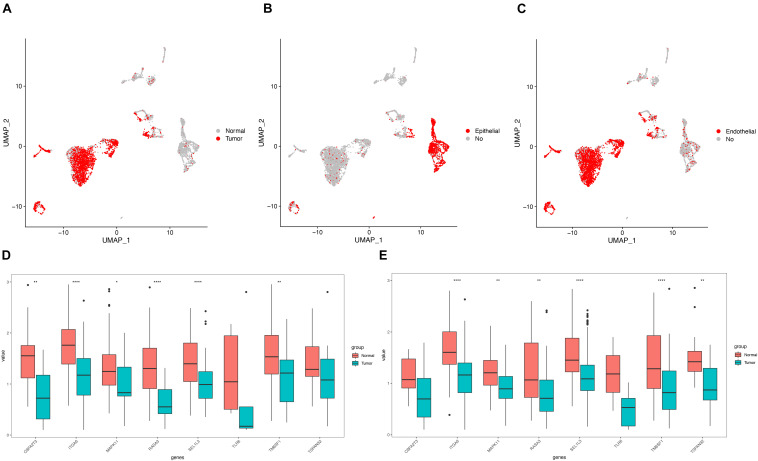
Validation of the expression by single cell RNA-seq. **(A)** Clustering of tumor and non-tumor cells using Uniform Manifold Approximation and Projection (UMAP); UMAP showing the epithelial and non- epithelial cells **(B)**; endothelial and non-endothelial cells **(C)**; The expression of 9 hub genes in the epithelial cells **(D)**, and epithelial cells **(E)** from tumor and non-tumor.

To understand heterogeneous cell populations and measure the cell-to-cell expression variability of thousands of genes, single-cell RNA-sequencing has emerged as a powerful method to perform transcriptome profiling at a single-cell level. We downloaded and analyzed the single-cell transcriptome data from two patients with LUAD from GSE149655. Cluster-specific genes were used to annotate cell types with classic markers described in previous studies (Lambrechts et al., 2018; Chen et al., 2020b): epithelial (CAPS, KRT8, and KRT18) and endothelial (CLDN5, FCN3, and RAMP2). The analysis identified different clusters of tumor and non-tumor cells (Figure 8A), epithelial and non-epithelial (Figure 8B), and endothelial and non-endothelial cells (Figure 8C). Notably, the expression of nine key genes in the epithelial cells (Figure 8D) and epithelial cells from tumor and non-tumor (Figure 8E) cells were nearly consistent with the levels of mRNA by RNA-sequencing from TCGA (Figure 7A). The expression of each gene is presented in Supplementary Figure 4.

### Prognostic and Clinicopathological Differences Between High-Risk and Low-Groups

**FIGURE 9:**
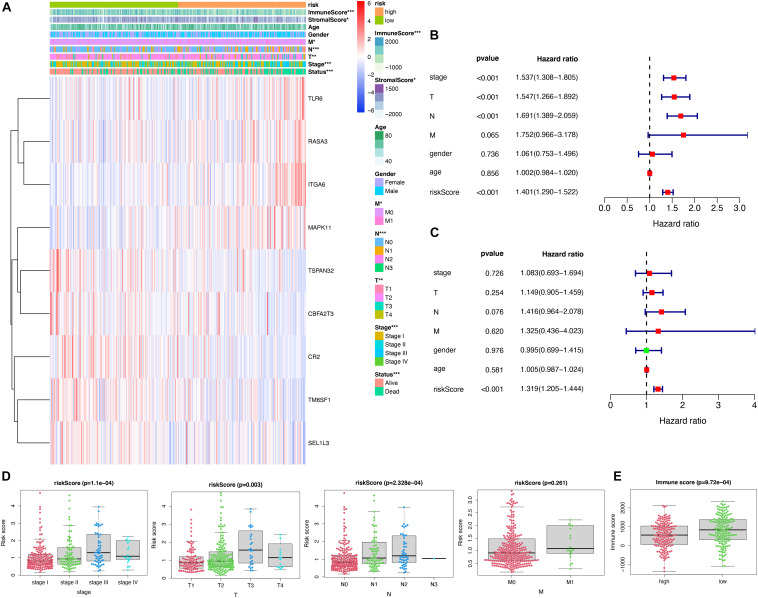
Clinical significance and the expression of hub genes in risk groups. **(A)** The expression of 9 hub genes in the Heatmap and clinicopathologic features of high- and low-risk groups; **(B)** Univariate Cox analysis was used to evaluate the prognostic value of common clinical parameters and hub genes; **(C)** Multivariate Cox analysis was conducted to evaluate the prognostic value of common clinical parameters and hub genes; **(D)** Distribution of risk scores stratified by stages, T, N, and M; **(E)** the immunoscore in high- and low-risk groups **(D)**. *p < 0.05, **p < 0.01, and ***p < 0.001.

A heatmap was presented to reveal the differences describing clinicopathological features and the levels of nine genes. Strikingly, the high-risk group was strongly correlated with immunoscore, stromalscore, the M, N, T, TNM stage, and status (Figure 9A). The univariate Cox analysis indicated the TNM stage, T, N, M, and risk score were significantly associated with survival (Figure 9B). However, the multivariate Cox regression model showed the risk score (*P* < 0.001, HR = 1.319, 95% CI = 1.205–1.444) was the only independent prognostic factor for LUAD (Figure 9C). Meanwhile, the association between the risk scores and several clinicopathological features were explored. Patients with an advanced TNM stage (*P* = 1.1E-0.4), T (*P* = 0.003) and N (*P* = 2.328E-0.4) tended to have higher risk scores (Figure 9D). Strikingly, the immune scores were significantly higher for the high-risk group than those of the low-risk group (Figure 9E), suggesting that the risk model might play a vital role in the process of the tumor microenvironment.

### Nomogram Construction

**FIGURE 10:**
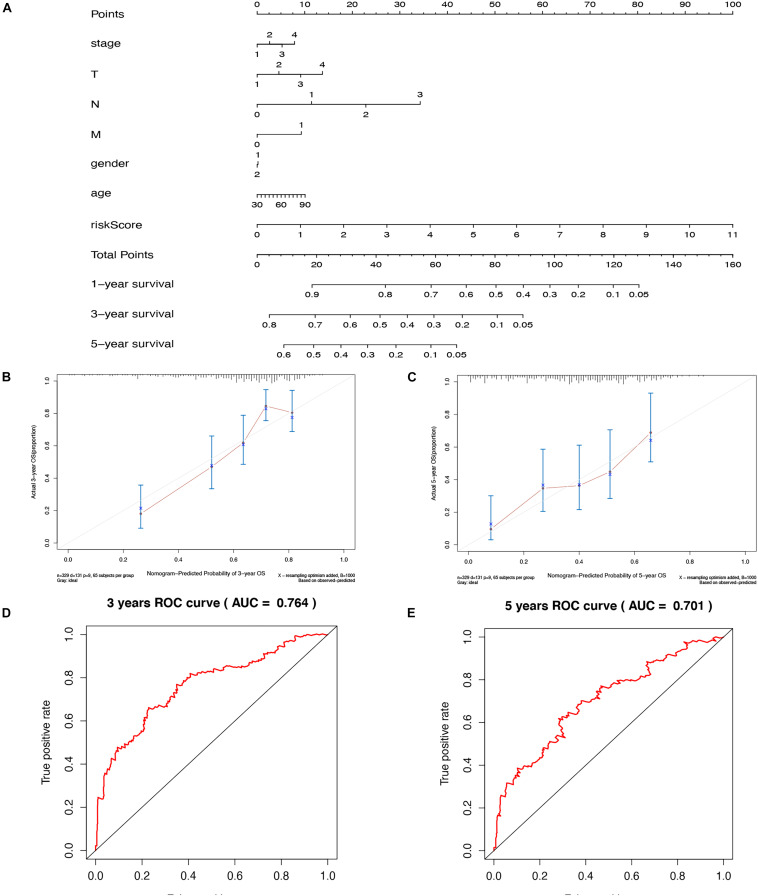
Nomogram and calibration plots of the prognostic model. **(A)** Nomogram to predict 1-year, 3-year, and 5-year OS in the TCGA cohort; Calibration plots of the nomogram to predict OS at 3 years **(B)** and 5 years **(C)**; ROC curves and AUC for 3-year **(D)**, and 5-year **(E)** The survival of the nomogram.

To establish a clinical strategy to predict the survival probability with LUAD patients, a nomogram was created based on the TCGA cohort to evaluate the probability of the 3- and 5-year OS. The predictors of the nomogram contained seven prognostic factors including stage, T, N, M, gender, age, and risk score (Figure 10A). The calibration curves for the 3-year and 5-year OS rates uncovered favorable consistency between the actual observation and predictive value (Figures 10B,C). Furthermore, the prediction accuracy of the nomogram was assessed using the ROC curve. The results revealed that the AUCs of the nomogram score for 3-year and 5-year were 0.764 and 0.701, respectively (Figures 10D,E). After a comprehensive assessment of prognostic value, the risk model was considered as predicted biomarkers for LUAD patients.

### Correlation Between Levels of Hub Genes and Immune Cell Infiltration

**FIGURE 11:**
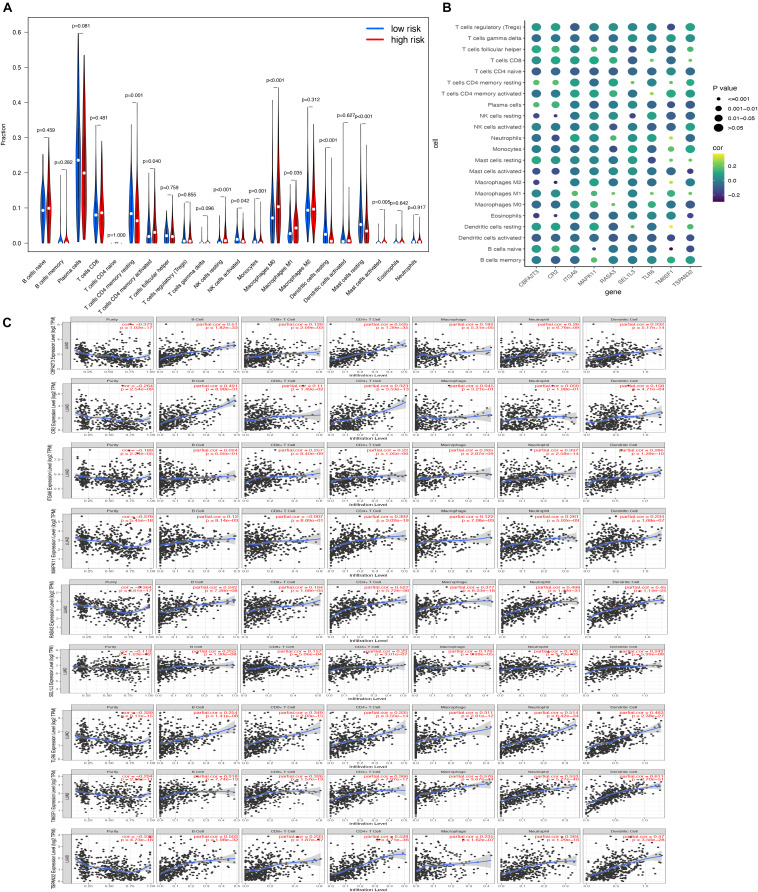
Association of hub genes’ expression with immune infiltration in LUAD. **(A)** The comparisons of 22 infiltrated immune cells between the high- and low-risk groups; **(B)** the correlation of every hub gene and 22 immune cell types. The point size represented P-value and shade of color represented Pearson correlation index r; **(C)** The relationship between the hub gene and the infiltration amount of six types of immune cells.

In order to determine the role of risk scores in tumor microenvironments, we finally explored the association between risk scores and 22 immune cells. Notably, activated CD4 memory T cells, resting NK cells, M0 macrophages, M1 macrophages, and activated mast cells were enriched in the samples of the high-risk group, while the samples in the low-risk group were significantly correlated with resting CD4 memory T cells, activated NK cells, monocytes, resting dendritic cells, and resting mast cells (Figure 11A). Additionally, it was indicated that all nine hub genes were associated with tumor purity, B cells, CD8 + T cells, CD4 + T cells, macrophages, neutrophil, and dendritic cells. The results showed a high correlation with the level of immune cell infiltration (Figures 11B,C).

### Strong Therapeutic Potential Shown by 13 Small Molecule Drugs

Highly associated molecule drugs were identified by CMap. In total, 13 molecule drugs were screened, including vorinostat, lynestreno, sulfamerazine, amiodarone, cefalexin, chlorpropamide, tetracycline, Fenbufen, cephaeline, diazoxide, vincamine, fluocinonide, and josamycin (Table 2), and they were identified as potential novel drug candidates for LUAD treatment.

**TABLE 2:** Potential small molecular drugs for LUAD patients.

Cmap name	Mean	*n*	Enrichment	*p*	Specificity	%non-null
Vorinostat	–0.553	12	–0.701	0	0.0885	91
Lynestreno	0.435	5	0.774	0.0013	0.0056	80
Sulfamerazine	0.464	5	0.772	0.00136	0	80
Amiodarone	0.458	5	0.768	0.0015	0.0069	80
Cefalexin	0.415	5	0.748	0.00236	0	80
Chlorpropamide	0.442	6	0.667	0.00381	0	83
Tetracycline	–0.525	5	–0.708	0.00457	0.0189	80
Fenbufen	0.402	6	0.611	0.01087	0	66
Cephaeline	0.439	5	0.619	0.02373	0.4795	80
Diazoxide	–0.555	5	–0.608	0.02597	0.0427	80
Vincamine	0.423	6	0.539	0.03693	0.162	66
Fluocinonide	0.413	5	0.584	0.03839	0.0746	80
Josamycin	–0.401	5	–0.562	0.04908	0.1	60

## Discussion

In our study, we identified 1,219 significant DEGs between LUAD tumors and normal tissues. Then a WGCNA was built, and nine hub genes was explored by univariate regression, LASSO and multivariate regression analysis. Moreover, the prognostic risk model, acting as an independent factor, was highly correlated with immune cell infiltration. A prognostic risk model including *CBFA2T3*, *CR2*, *SEL1L3*, *TM6SF1*, *TSPAN32*, *ITGA6*, *MAPK11*, *RASA3*, and *TLR6* serves as a novel biomarker and showed the prognostic and predictive values for LUAD.

We first analyzed the expression profiles from six high-quality GEO datasets to explore key genes related to LUAD. The most upregulated gene SPINK1 has been determined to promote cell growth and metastasis of LUAD, acting as a novel prognostic biomarker (Xu et al., 2018; Guo et al., 2019). Meanwhile, the most downregulated gene TMEM100 is minimally expressed in non-small cell lung cancer and enhances the sensitivity to chemotherapy in gastric cancer (Han et al., 2017; Zhuang et al., 2020), which is a finding highly similar to ours. There was a similar conclusion with our findings that the top dysregulated genes are distributed in chromosomes 3 (Song et al., 2019). After exploring the enrichment of the DEGs in GO and KEGG pathways, we found that DEGs might correlate with tumor development. The WGCNA allowed us to identify the co-expression module associated with clinical traits. In the module, genes were enriched in lymphocyte differentiation, the chemokine signaling pathway, and the B cell receptor signaling pathway, indicating the potential roles in tumor environments.

Based on the univariate Cox regression, LASSO, and multivariate Cox regression analyses, nine hub genes (*CBFA2T3, CR2, SEL1L3, TM6SF1, TSPAN32, ITGA6, MAPK11, RASA3, and TLR6*) were obtained to explore the prognostic value in LUAD. *CBFA2T3* (*MTG16*), CBFA2/RUNX1 Partner Transcriptional Co-Repressor 3, has not been clearly studied in lung cancer (Zhang et al., 2018) although the CBFA2T3-GLIS2 fusion transcript is well proven as a novel common feature in pediatric cytogenetically normal acute myeloid leukemia (AML) (Gruber et al., 2012; Masetti et al., 2013; Smith et al., 2020). In breast cancer, the expression of *CBFA2T3* is lower in normal breast tissue compared to the primary tumors, consistent with our finding in the clusters of epithelial and endothelial cells by analyzing single-cell RNA-sequencing data (Kochetkova et al., 2002). The receptor for complement C3 (*CR2*), a receptor for the Epstein-Barr virus on human B cells and T cells, activates B lymphocytes (Barel et al., 1995; Wu et al., 2002). Notably, genetic variations of *CR2* were associated with susceptibility to systemic lupus erythematosus type 9 (*SLEB9*), while the alteration rates of CR2 was highest as much as 3% for LUAD patients among nine hub genes in our work. The suppression of Lin-12-like protein (*SEL1L3*), has been demonstrated as a member of a prognostic signature and involved in the development of melanoma and immune response (Mei et al., 2021). *TM6SF1*, transmembrane 6 Superfamily Member 1, has been found to be a DNA promoter hypermethylation marker in breast cancer (de Groot et al., 2014, 2016). Meanwhile, the expression of *TM6SF1* in AML samples has been much higher than that in normal samples (Cheng et al., 2020). *TSPAN32* has been found as a potential tumor suppressor in Wilms tumors, while the expression was higher in tumor samples compared to normal tissues in our study (Charlton et al., 2015). It has been discovered that higher levels of Integrin alpha 6 (*ITGA6*) are expressed in the highly invasive pancreatic cancer cells than in less invasive cells, resulting in a poor prognosis in pancreatic patients via TCGA (Wu Y. et al., 2019). However, based on our data, there was no significant difference in the mRNA levels of *ITGA6* between tumor and normal tissues by TCGA and single-cell RNA-sequencing. Furthermore, there was a similar observation with our finding that mitogen-activated protein kinase 11 (*MAPK11*) was highly expressed in metastatic breast cancer patients and in the breast cancer cell lines (He et al., 2014). RAS P21 Protein Activator (*RASA3*) has been determined to be a novel tumor suppressor with low expression in colorectal and bladder tumor (Yao et al., 2007; Tang et al., 2014). Of note, we additionally identified low expression in LUAD tumor samples. Furthermore, a decrease of TLR6 expression in colorectal tumor samples has been found compared to normal colon tissues. Thus, the nine hub genes examined in our study are key biomarkers in the development and prognosis of cancer, which has been supported by several previous findings, including on colorectal cancer, AML, and melanoma.

To further explore the prognostic value of identified genes, a risk model was established depending on the expression of key genes. After a comprehensive analysis of clinical features, the patients in the high-risk group had a less positive overall survival and had advanced tumor stages, which indicated the prognostic model was highly reliable for prognosis prediction. Interestingly, the immune scores were much higher in the high-risk group. It validated our hypothesis that the risk model consisting of nine hub genes might be closely associated with tumor microenvironments. The nomogram developed in our study showed an ideal performance in OS prediction at three and five years.

Increasing attention has been paid to tumor microenvironments, including immune cell infiltration, in recent decades. We found that high-risk-score LUAD patients had higher proportions of activated CD4 memory T cells, resting NK cells, M0 macrophages, M1 macrophages, and activated mast cells, confirming that the risk model had a regulatory effect on tumor microenvironments. In addition, all nine hub genes were highly associated with B cells, CD8 + T cells, CD4 + T cells, macrophages, neutrophil, and dendritic cells, which provided a possible use for cancer immunotherapy. Strikingly, tumor-infiltrating immune cells in lung cancer are likely to be important determinants of both the prognosis and response to immunotherapies (Bremnes et al., 2016; Liu et al., 2017; Muppa et al., 2019). However, the intricate mechanisms of the new biomarkers and immune cells need to be explored in further experiments.

Concerning the vital roles of hub genes and the risk model in the prognosis and prediction of LUAD, we assessed possible small molecular drugs depending on the expression of genes using CMap. Vorinostat is a histone deacetylase inhibitor, approved to treat cutaneous T-cell lymphoma (Olsen et al., 2007; Kim et al., 2018). Simultaneously, Pembrolizumab plus vorinostat have demonstrated preliminary antitumor activity in advanced non-small cell lung cancer and metastatic head and neck squamous cell carcinomas in clinical trials, which is highly consistent with our assessment (Gray et al., 2019; Rodriguez et al., 2020). Although several assessed drugs in our study did not show a clear effect on previous cancers, there might be a certain value in the combination of other anti-LUAD drugs.

Nevertheless, several limitations need to be noted in our work. First, even though we enrolled as many patients as possible according to the inclusion criteria, more samples may enhance the confidence levels of our conclusions. Moreover, subsequent fundamental researches are required to validate and corroborate the results *in vitro* and vivo. Third, the interaction between the nine hub genes and the potential mechanisms in LUAD has not been explored clearly and should be examined in the future.

## Conclusion

In summary, we identified nine hub genes highly associated with the progression of LUAD. A prognostic risk model established for the nine key genes was validated as an independent factor for LUAD and highly correlated with immune infiltrating, which suggested potential guidance for LUAD prognosis and molecular targeted therapy. However, further biological explorations are required to prove the functions of the predictive genes in the progression of LUAD.

## Data Availability Statement

Publicly available datasets were analyzed in this study. The names of the repository/repositories and accession number(s) can be found in the article/Supplementary Material.

## Author Contributions

YS designed the study. HZ performed data analyses and wrote the manuscript. JW designed analysis strategies. YZ helped prepare for the manuscript. All authors read and approved the final manuscript.

## Conflict of Interest

The authors declare that the research was conducted in the absence of any commercial or financial relationships that could be construed as a potential conflict of interest.

## Publisher’s Note

All claims expressed in this article are solely those of the authors and do not necessarily represent those of their affiliated organizations, or those of the publisher, the editors and the reviewers. Any product that may be evaluated in this article, or claim that may be made by its manufacturer, is not guaranteed or endorsed by the publisher.

## Footnotes

1
http://cbioportal.org

2
https://cistrome.shinyapps.io/timer/

## Supplementary Material

Supplementary Figure 1 Volcano plot visualizing DEGs between LUAD tumor and normal tissues in **(A)** GSE43458, **(B)** GSE62949, **(C)** GSE68465, **(D)** GSE115002, **(E)** GSE116959, and **(F)** GSE118370 datasets.

Supplementary Figure 2 DEGs were analyzed by LASSO regression analysis. **(A)** LASSO-Cox regression coefficient selection and variable screening. The lower horizontal axis represents lambda value, and the upper horizontal axis scale represents the number of variables in the lasso-cox regression model, the regression coefficient (x) of which is not 0. The left vertical axis represents the value of the regression coefficient (x); **(B)** Cross-validation in the LASSO-cox regression model to select the tuning parameter. The horizontal axis represents the log (lambda) value, and the vertical axis represents partial likelihood deviance. The red dots in the figure represent partial likelihood deviations ± standard error for different tuning parameters.

Supplementary Figure 3 The validation of the risk model in an independent GEO dataset (GSE68465).

Supplementary Figure 4 The correlation of the expression of each hub gene with TNM stage.

Supplementary Figure 5 UMAP presenting the expression of each gene in tumor and normal tissues by single cell RNA-seq.
