# Supplementary figures and images for: Comprehensive Analysis of a Nine-Gene Signature Related to Tumor Microenvironment in Lung Adenocarcinoma
Source: Front Cell Dev Biol. 2021 Sep 1;9:700607. doi: 10.3389/fcell.2021.700607 (PMC8440811; doi:10.3389/fcell.2021.700607)

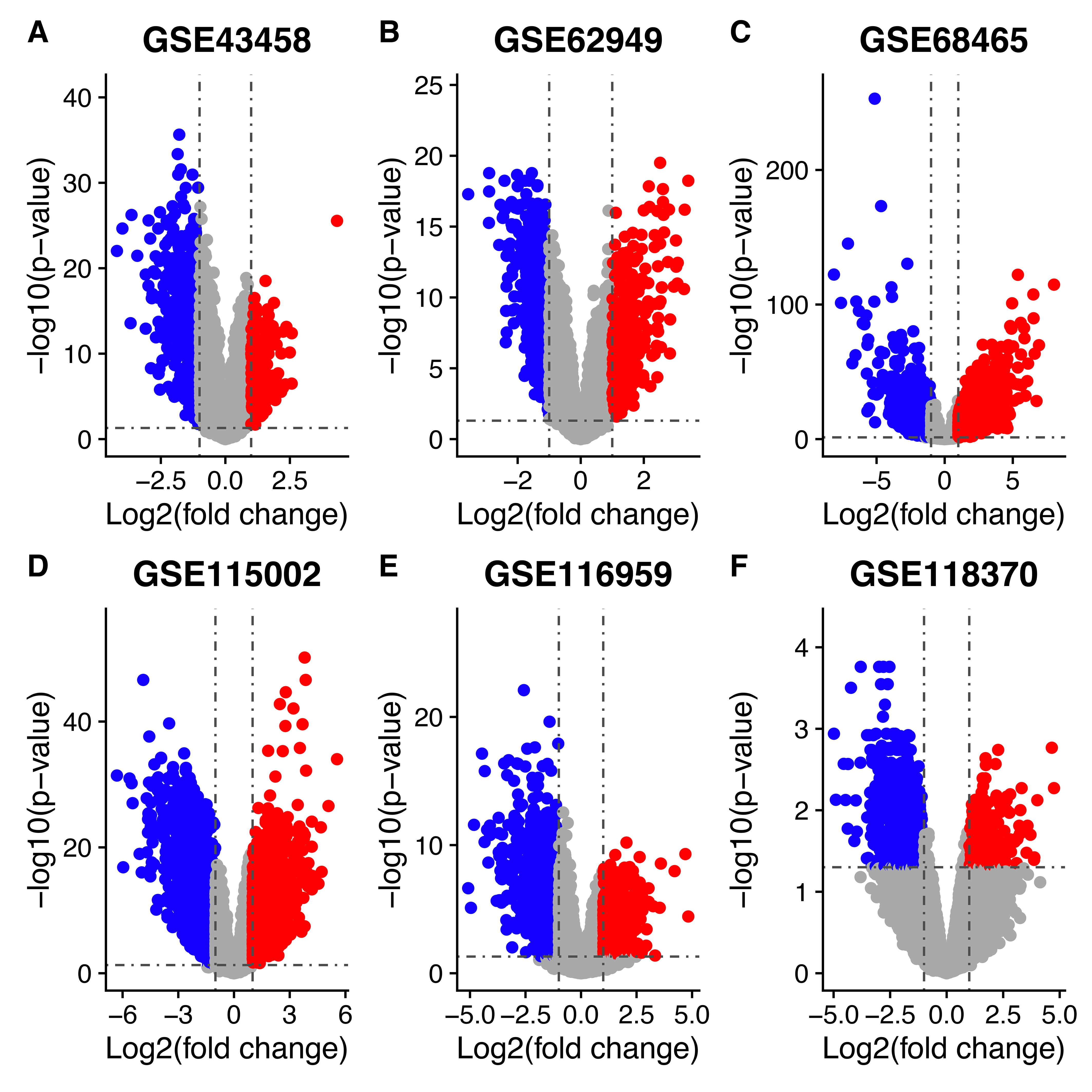

Supplement: Supplementary Figure 1 — Volcano plot visualizing DEGs between LUAD tumor and normal tissues in (A) GSE43458, (B) GSE62949, (C) GSE68465, (D) GSE115002, (E) GSE116959, and (F) GSE118370 datasets. [file Image_1.TIF]

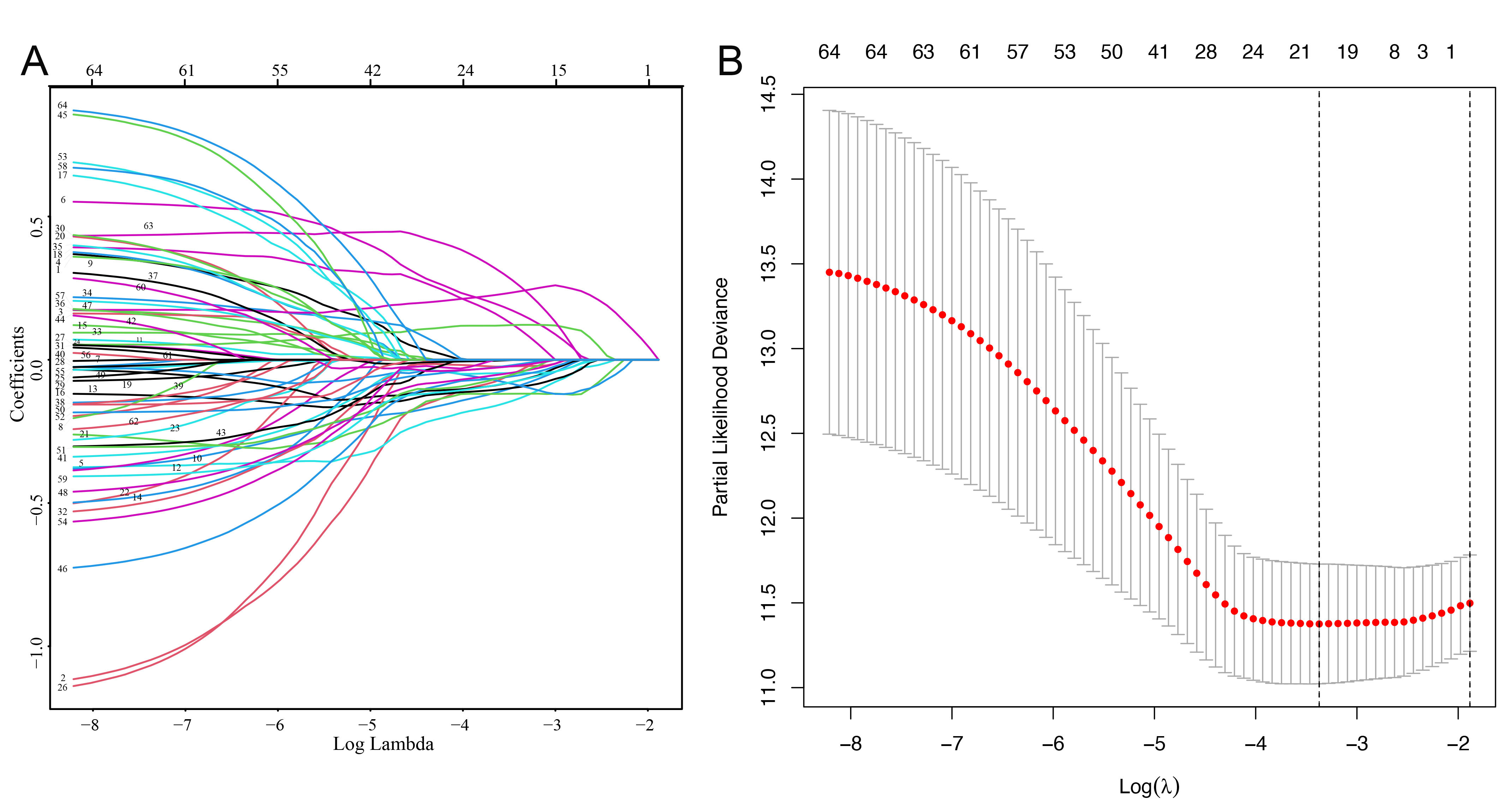

Supplement: Supplementary Figure 2 — DEGs were analyzed by LASSO regression analysis. (A) LASSO-Cox regression coefficient selection and variable screening. The lower horizontal axis represents lambda value, and the upper horizontal axis scale represents the number of variables in the lasso-cox regression model, the regression coefficient (x) of which is not 0. The left vertical axis represents the value of the regression coefficient (x); (B) Cross-validation in the LASSO-cox regression model to select the tuning parameter. The horizontal axis represents the log (lambda) value, and the vertical axis represents partial likelihood deviance. The red dots in the figure represent partial likelihood deviations ± standard error for different tuning parameters. [file Image_2.TIF]

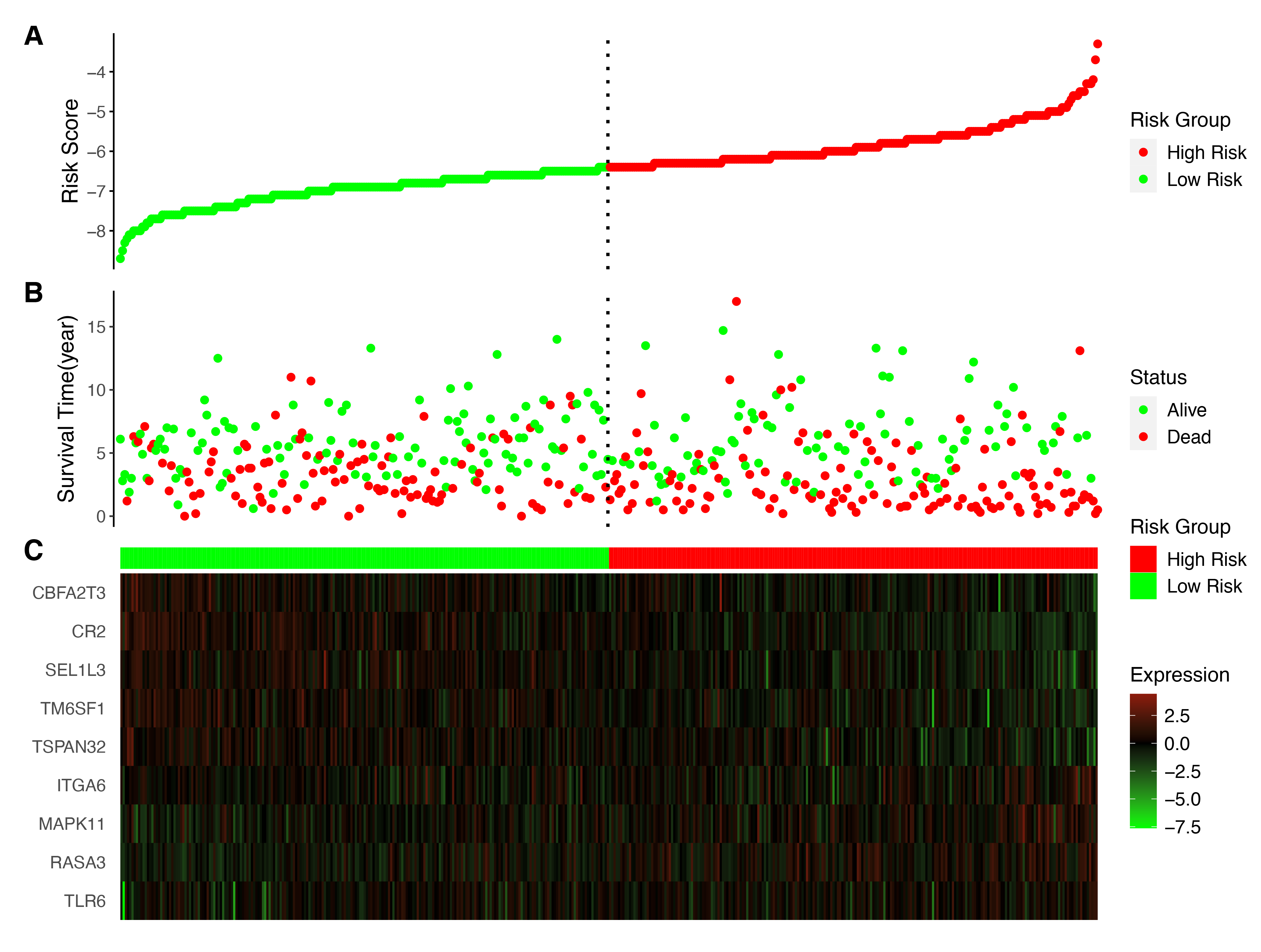

Supplement: Supplementary Figure 3 — The validation of the risk model in an independent GEO dataset (GSE68465). [file Image_3.TIF]

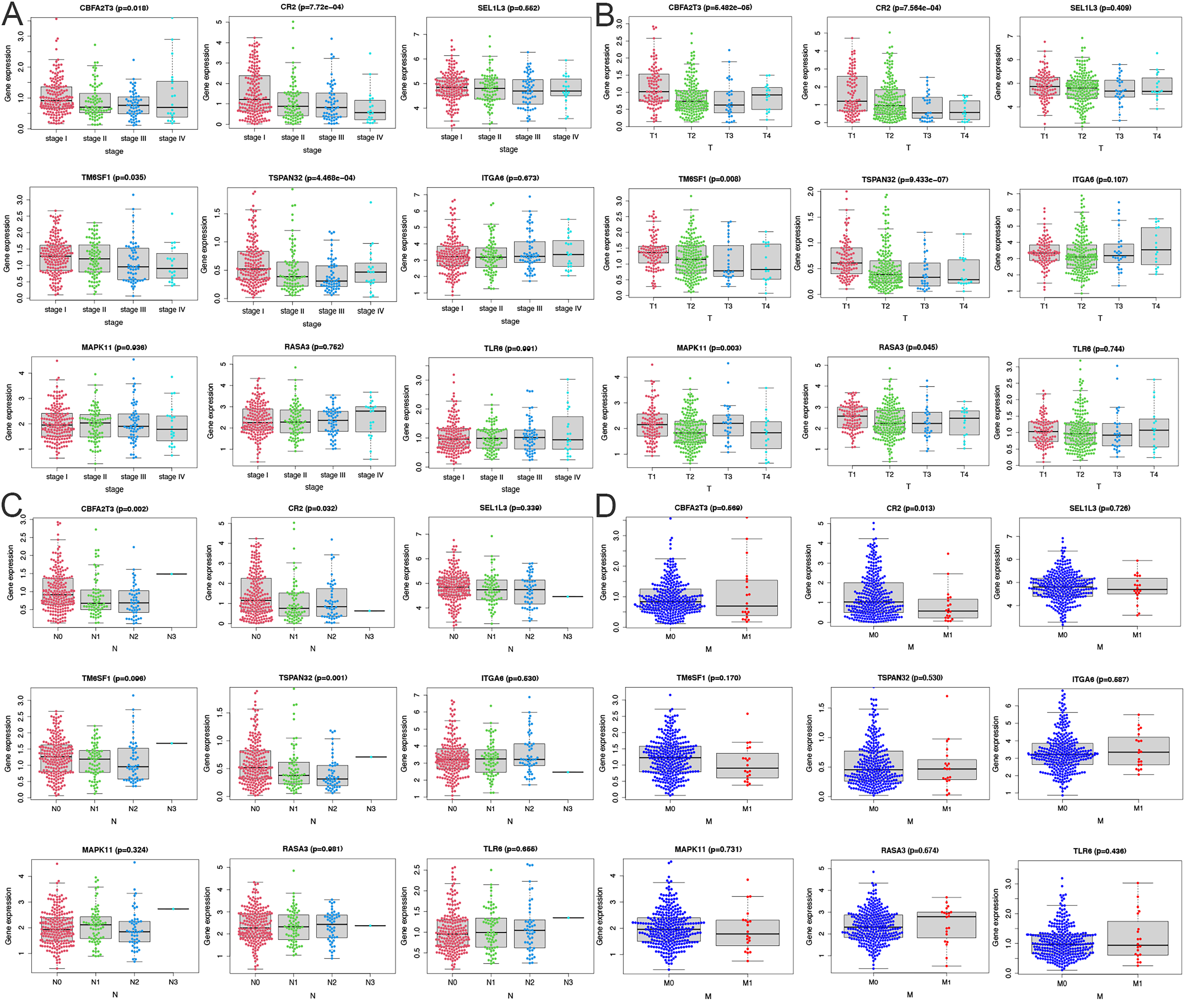

Supplement: Supplementary Figure 4 — The correlation of the expression of each hub gene with TNM stage. [file Image_4.TIF]

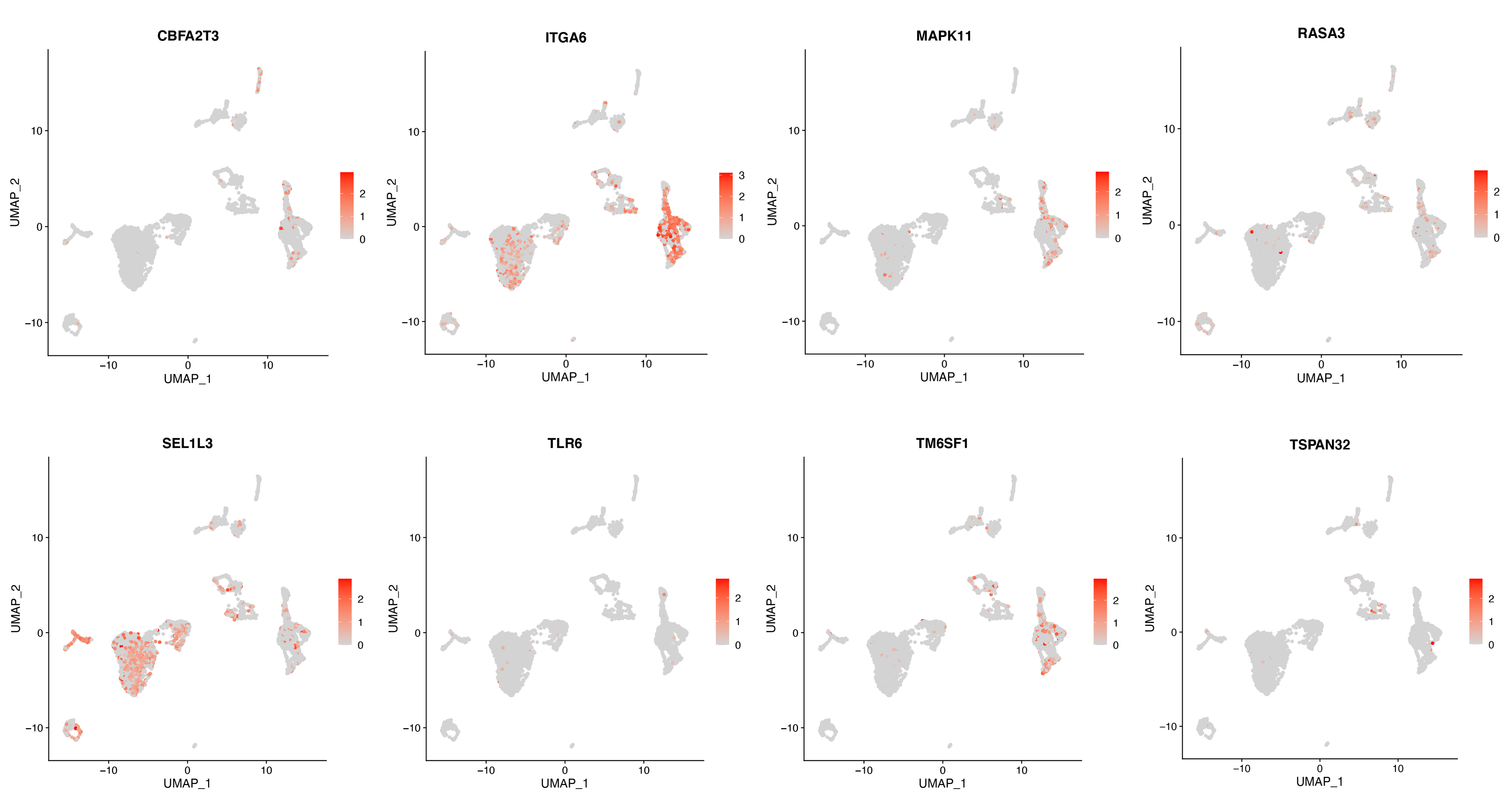

Supplement: Supplementary Figure 5 — UMAP presenting the expression of each gene in tumor and normal tissues by single cell RNA-seq. [file Image_5.TIF]
